# Effects of Manipulated Above- and Belowground Organic Matter Input on Soil Respiration in a Chinese Pine Plantation

**DOI:** 10.1371/journal.pone.0126337

**Published:** 2015-05-13

**Authors:** Juan Fan, Jinsong Wang, Bo Zhao, Lianhai Wu, Chunyu Zhang, Xiuhai Zhao, Klaus v. Gadow

**Affiliations:** 1 Key Laboratory for Silviculture and Conservation of the Ministry of Education, Beijing Forestry University, No. 35 Tsinghua East Road, Haidian District, Beijing 100083, P. R. China; 2 Rothamsted Research, North Wyke, Okehampton, Devon EX20 2SB, United Kingdom; 3 Faculty of Forestry and Forest Ecology, Georg-August-University Göttingen, Büsgenweg 5, D-37077 Göttingen, Germany; Fudan University, CHINA

## Abstract

Alteration in the amount of soil organic matter input can have profound effect on carbon dynamics in forest soils. The objective of our research was to determine the response in soil respiration to above- and belowground organic matter manipulation in a Chinese pine (*Pinus tabulaeformis*) plantation. Five organic matter treatments were applied during a 2-year experiment: both litter removal and root trenching (LRRT), only litter removal (LR), control (CK), only root trenching (RT) and litter addition (LA). We found that either aboveground litter removal or root trenching decreased soil respiration. On average, soil respiration rate was significantly decreased in the LRRT treatment, by about 38.93% ± 2.01% compared to the control. Soil respiration rate in the LR treatment was 30.65% ± 1.87% and in the RT treatment 17.65% ± 1.95% lower than in the control. Litter addition significantly increased soil respiration rate by about 25.82% ± 2.44% compared to the control. Soil temperature and soil moisture were the main factors affecting seasonal variation in soil respiration. Up to the 59.7% to 82.9% seasonal variation in soil respiration is explained by integrating soil temperature and soil moisture within each of the various organic matter treatments. The temperature sensitivity parameter, *Q*_10_, was higher in the RT (2.72) and LA (3.19) treatments relative to the control (2.51), but lower in the LRRT (1.52) and LR treatments (1.36). Our data suggest that manipulation of soil organic matter input can not only alter soil CO_2_ efflux, but also have profound effect on the temperature sensitivity of organic carbon decomposition in a temperate pine forest.

## Introduction

Globally, it is assumed that soils store more organic carbon than carbon in plants and the atmosphere combined [1]. Temperate forest soils contain more than twice as much carbon as the living biomass [2]. As a result, changes in the dynamics of soil carbon of temperate forests could have a profound impact on global carbon cycling. Soil respiration in forest ecosystems is a major pathway for carbon dioxide (CO_2_) returning to the atmosphere. It originates from the decay of organic matter in the soil, the decomposition of aboveground litter and respiration within the rhizosphere, including roots and mycorrhizae.

Soil respiration can be affected by soil microclimatic factors, such as temperature, soil moisture and pH [3]. Increasing evidence also suggests there is a strong linkage between soil respiration and recent photosynthates [4]. As a biological process, soil respiration is closely tied to plant growth and the supply of photosynthetic substrates. Tree girdling experiments [5], shading and clipping experiments [6] and correlations between soil respiration and the supply of substrates among different forest sites [7], have all been cited as evidence for above- and belowground organic matter input being the main driver of soil respiration. Therefore, any factor that impacts the above- and belowground substrate supply is likely to cause changes in soil respiration and corresponding climate-carbon feedbacks.

In the context of global change, alterations in the amount of aboveground litter are becoming more and more likely. It has been reported that elevated atmospheric CO_2_ concentrations [8], increased nitrogen deposition [9] and rising temperatures are predicted to raise the amount of aboveground litter via enhanced plant productivity, whereas elevated O_3_ [10] and drought stress [11] generally decrease productivity. Some other drastic disturbances, i.e., severe ice storms [12], insect and disease infestation [13] and wildfires [14] can also lead to sudden and dramatic changes in litter input. In addition, human activity such as litter layer removal and understory clearing are common forest management practices in many regions [15,16,17,18], with the aim not only to harvest fuel, but also to eliminate combustible loads to prevent fire. These changes in the amount of aboveground litter can inevitably lead to a direct effect on soil respiration, via changes in microclimate of the litter layer and alterations in the supply of substrates.

Changes in the amount of soil organic matter are closely related to physical, chemical and biological processes in the soil [19], but the underlying mechanisms controlling the effect of soil organic matter input on soil respiration are still not well understood. Organic matter manipulation involving litter removal or addition is a direct way for studying the effect of the amount of litter on soil respiration [20]. However, existing evidence on the effect of soil organic matter on soil respiration may differ greatly among forest ecosystems. It has been reported that litter removal often drives proportional declines in soil respiration [21,22,23,24]. Soil respiration may increase disproportionately in response to litter addition, suggesting that increased litter input may not only release a portion of the newly added carbon, but also accelerate the decomposition of older organic matter through a priming effect [25].

Soil respiration is not only affected by the amount of aboveground litter, but also influenced by the supply of organic matter input to soil through root turnover and root exudates. Roots play an important role in translocating photosynthates from plants to the soil [26]. Microbial activity may increase with the decomposition of root debris [27]. In addition, roots release exudates including carbohydrates, sugars, amino acids, organic acids and phenolic compounds [28]. It has been estimated that approximately 75% of carbon allocated to the roots is respired by soil microorganisms [29]. The contribution of root respiration to total soil respiration can account for as little as 10% to more than 90% worldwide [30]. It has been reported that the absence of roots eliminates the supply of root exudates and may decrease microbial activity, which in turn inhibits soil CO_2_ efflux [26]. Although this effect is of ecological importance, changes in belowground roots have usually been ignored in ecological studies of forests.

In this study, we use an *in situ* above- and belowground organic matter manipulation experiment in a Chinese pine (*Pinus tabulaeformis*) plantation in order to test our hypothesis that (1) either aboveground litter removal or root trenching will always decrease soil respiration, whereas litter addition will increase it; (2) litter removal will have a higher impact than root trenching on soil respiration through our entire observation period because roots might be able to maintain respiration for some time after root trenching; (3) the extent of increase in soil respiration in the litter addition treatment will be larger than that of decrease in soil respiration only in the litter removal treatment because of the priming effect.

## Materials and Methods

### Ethics statement

The research station for this study is owned by Beijing Forestry University. Our study was approved by the Taiyue Mountain Ecosystem Research Station and the Key Laboratory for Silviculture and Conservation of the Ministry of Education.

### Site description

Field work was carried out at the Taiyue Mountain Ecosystem Research Station (36°18´ N, 111°45´ E, 1560 m a.s.l), located in Shanxi Province, in northern China (the Taiyue Forestry Bureau issued the permission to conduct this study at each location). The region is classified as belonging to a warm-temperate semi-arid continental monsoon-affected climate, with a mean annual temperature of 9.9°C. The highest monthly average temperature of 22.4°C is observed in July while the lowest monthly average temperature of-4.6°C occurs in January. Mean annual precipitation is 548 mm with a mean relative humidity of 65%. The distribution of precipitation over a year is relatively uneven. The wet season is from July to September and accounts for more than 60% of annual precipitation. The soil is a typical brown forest soil of 60–100 cm in thickness. The soil pH ranges from 6.8 to 7.3 and soil organic carbon is 3%– 4%.

The study field is an artificial forest dominated by a 38-year *Pinus tabulaeformis* stand that has been protected since the 1990s. Stand density is 2213 stems per hectare. The dominant overstory vegetation in the stand is *P*. *tabulaeformis* with a mean breast-height diameter of 14.5 cm ± 1.5 cm and a mean height of 16.8 m ± 1.8 m. The understory layer consists mainly of *Ostryopsis davidiana*, *Lespedeza bicolor*, *Hippophae rhamnoides*, *Corylus mandshurica*, *Swida bretchneideri* and *Rosa xanthina*. Mean annual litterfall in the forest is 504 g·m^-2^ and the density of fine roots is 192 g·m^-2^.

### Experimental layout

Three 30×30 m plots were established in the plantation in January 2010. All live and dead trees with woody stems exceeding 1 cm in diameter at their breast-height were tagged and identified in each plot. The breast-height diameter, tree height and crown dimension of each tree were measured and recorded. In order to evaluate soil conditions in the plantation, we randomly collected twenty soil samples with a volume of 100 cm^3^ in each plot from the top soil (0–20 cm) in March 2010. All plot samples were collected as one common sample and sieved to 2 mm to remove coarse fragments and then air-dried to analyze their bulk density and nutrient contents. The total amount of N was measured using Kjeldahl’s digestion with a salicylic acid modification [31], while total amount of phosphorus and potassium were fused using the NaOH method[32]. Soil organic carbon was measured following the method described by Kalembasa and Jenkinson [33].

**Fig 1:**
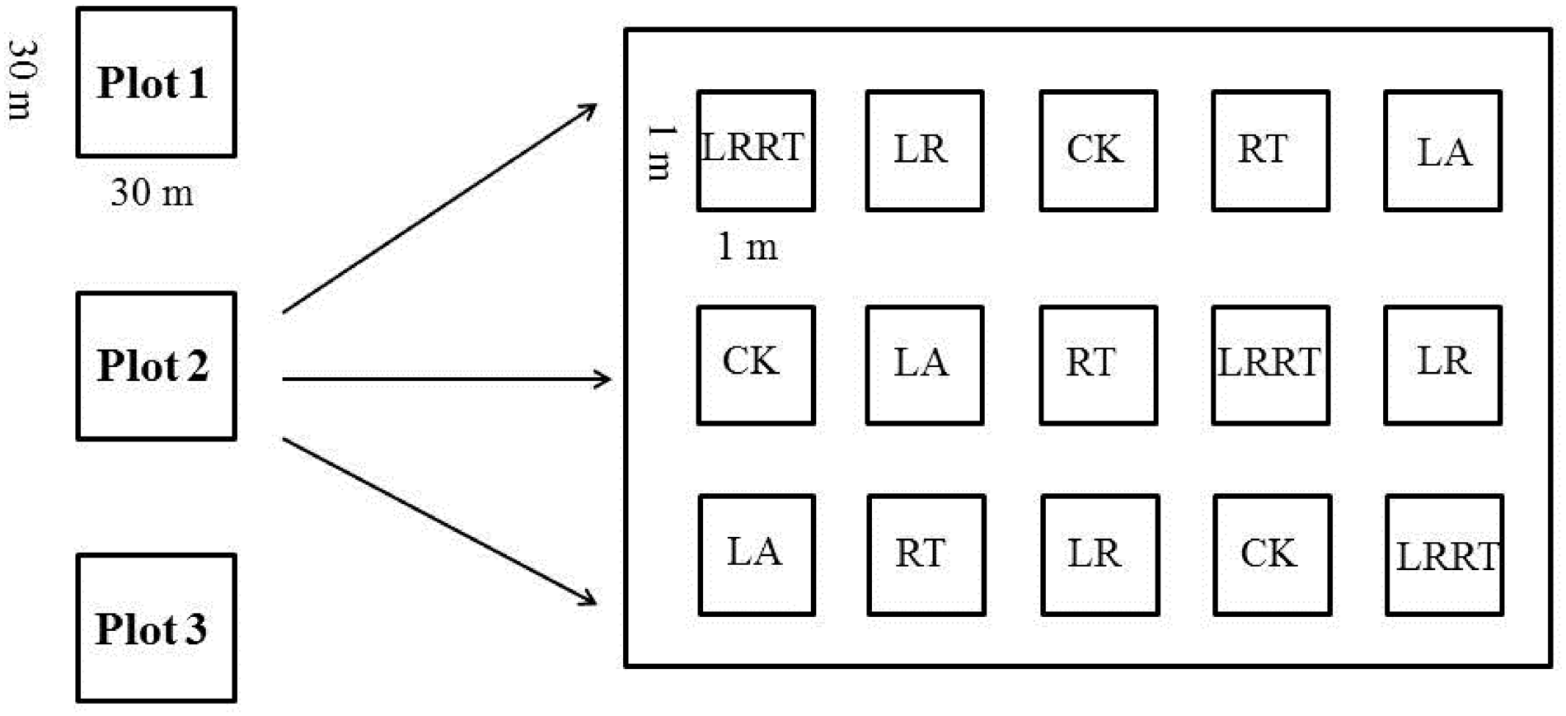
Schematic diagram of experimental plot layout. Organic matter treatments: both litter removal and root trenching (LRRT), only litter removal (LR), control (CK), only root trenching (RT), and litter addition (LA).

By the end of May 2010, fifteen 1×1 m subplots were randomly established in each plot for soil respiration measurements. The schematic diagram of the experimental plot layout is shown in Fig 1. The subplots were subjected to five organic matter treatments: (1) both litter removal and root trenching (LRRT for short), (2) only litter removal (LR), (3) control (CK), (4) only root trenching (RT), (5) litter addition (LA). Each treatment was replicated three times. In the LRRT and LR treatments, organic layers above the mineral soil were removed and a 1×1 m litter trap was set up 1 m above the ground to intercept fresh foliage. Root trenching was achieved by digging the subplot perimeter. To prevent roots originating from the plants growing outside of a subplot from penetrating into the subplot, a 0.5 mm thick polyethylene sheet along the sides of the trench was inserted before backfilling. The root-free plots were kept free of vegetation by cutting plant regrowth manually throughout the study period, with extra care taken to minimize disturbance to the soil. For the LA treatment, litter was transferred from the LR subplots. In each subplot, we inserted a 10 cm height cylinder with a 20 cm inner diameter into the soil up to 5–6 cm deep. We initiated soil respiration rate (*R*_s_) measurements 24 hours after installing the cylinder. The location of the cylinder did not change during the *R*_s_ measurements. We performed the *R*_s_ measurements in the middle and at the end of each month during the two growing seasons 2010 (from June to October) and 2011 (from May to October).

### Soil respiration rate, soil temperature and soil moisture measurements

We measured *R*_s_ from 8:00 to 18:00 on each day of the two measurements every month, using a portable, closed dynamic chamber (LI-8100, LI-COR, Nebraska, USA). Daily respiration rates were averaged for the calculation of accumulative monthly mean soil respiration efflux (g C m^-2^) for the various organic matter treatments. Accumulative monthly mean soil respiration efflux was calculated as follows:

$$CR_{s}=\left[DR_{s}\times \left(30/31\right)\times 24\times 3600\times 12\right]\cdot 10^{-6}$$ (1)

where *CR*_s_ is the accumulative monthly mean soil respiration efflux, *DR*_s_ is the daily mean soil respiration rate (μ mol m^-2^ s^-1^), 30/31 are the days of one month, 24 represents the number of hours per day, 3600 the number of seconds per hour and 12 is the molar mass of carbon (C). We summed *CR*_s_ to obtain the accumulative seasonal soil respiration efflux.

We also recorded soil temperature and soil moisture at 5 cm depth with the LI-8100 system simultaneously with the *R*_s_ measurements. In addition, air temperature and precipitation were measured using a Davis Weather Station (Vantage Pro, Davis Inc. USA) located at the Research Station.

### Modeling soil respiration rate with soil temperature and moisture

We used univariate and bivariate models to describe the relationship between soil respiration rate (*R*_s_), soil temperature (*T*) and soil moisture (*M*). The first model shows *R*_s_ as an exponential function with only soil temperature, *T* (°C, referred to as the *T* model) as the independent variable:

$$R_{s}=\beta_{0}e^{\beta_{1}T}$$ (2)

*R*_s_ as a function of soil moisture, *M* (%) was estimated with a quadratic equation (referred to as the *M* model):

$$R_{s}=\beta_{2}+\beta_{3}M+\beta_{4}M^{2}$$ (3)

In the third model, *R*_s_ is a function of both soil temperature and soil moisture (referred to as the *T & M* model):

$$R_{s}=\beta_{5}e^{\beta_{6}T}M^{\beta_{7}}$$ (4)

where *R*_s_ is the soil respiration rate (μ mol m^-2^ s^-1^); *T* the soil temperature at 5 cm depth (°C) and *M* is the volumetric water content of soil at a depth of 5 cm (%). As suggested by Lloyd and Taylor [34], soil temperature sensitivity, *Q*_10_, was calculated as follows: $Q_{10}=e^{10\beta_{1}}$, where *β*_1_ is taken from the *T* model (Eq 2; *β*_1_) and from the *T & M* model (Eq 4; *β*_6_)

### Soil respiration components

Soil respiration components were calculated according to the method proposed by Rey et al. [35] as follows:

$$R_{m}=R_{1};R_{L}=R_{3}–R_{2};R_{r}=R_{3}–R_{4}$$

where *R*_m_ is the heterotrophic respiration rate derived from the decomposition of soil organic matter in the soil; *R*_L_ the respiration rate from litter decomposition and *R*_r_ the rate of root respiration. *R*_1_, *R*_2_, *R*_3_ and *R*_4_ are, respectively, the soil respiration rates from the LRRT, LR, CK and RT subplots. To test the precision of our *R*_s_ measurements, the sum of *R*_m_, *R*_L_ and *R*_r_ was compared with the soil respiration rate in the CK subplots.

### Microbial biomass carbon measurements in the soil

In order to evaluate microbial biomass carbon in the soil of the various organic matter treatments, we randomly collected three soil cores with a 2.5 cm diameter from the top soil (0–10 cm) in each subplot in November 2010 and then mixed them to form a composite sample. After removing roots and plant residue, these composite samples were immediately sieved through a 2 mm mesh sieve in the field and kept refrigerated after transport to the laboratory. Microbial biomass carbon (MBC) in the soil was measured using a chloroform direct-fumigation extraction method [36] in the laboratory.

### Statistical analysis

The objective of the statistical analysis was to determine differences in *R*_s_ among organic matter treatments by fluctuation, i.e.,: fluctuation (%) = 100 (*A*–*B*)/*B*, where *A* represents *R*_s_ in the LRRT, LR, RT and LA treatments and B the corresponding value in the CK.

The effect of organic matter treatment, temporal (month-to-month) variation and their interactions on *R*_s_, *T* and *M* were analyzed using a two-way analysis of variance (ANOVA). Levene’s test was used to test for the homogeneity of variance. Two-way ANOVA was also used to examine the effects of aboveground litter and belowground roots on *R*_s_, *T* and *M* during the entire observation period. In addition, one-way ANOVA was conducted to analyze the significance of the difference in accumulative seasonal soil respiration efflux among the five organic matter treatments. Differences among treatments were compared by a multiple LSD test. The significant level was set at 0.05 and all statistical tests were performed using SPSS (ver. 16.0) and R 2.15.2 (http://www.R-project.org/).

## Results

### Effects of organic matter treatments on soil respiration rate

**Fig 2:**
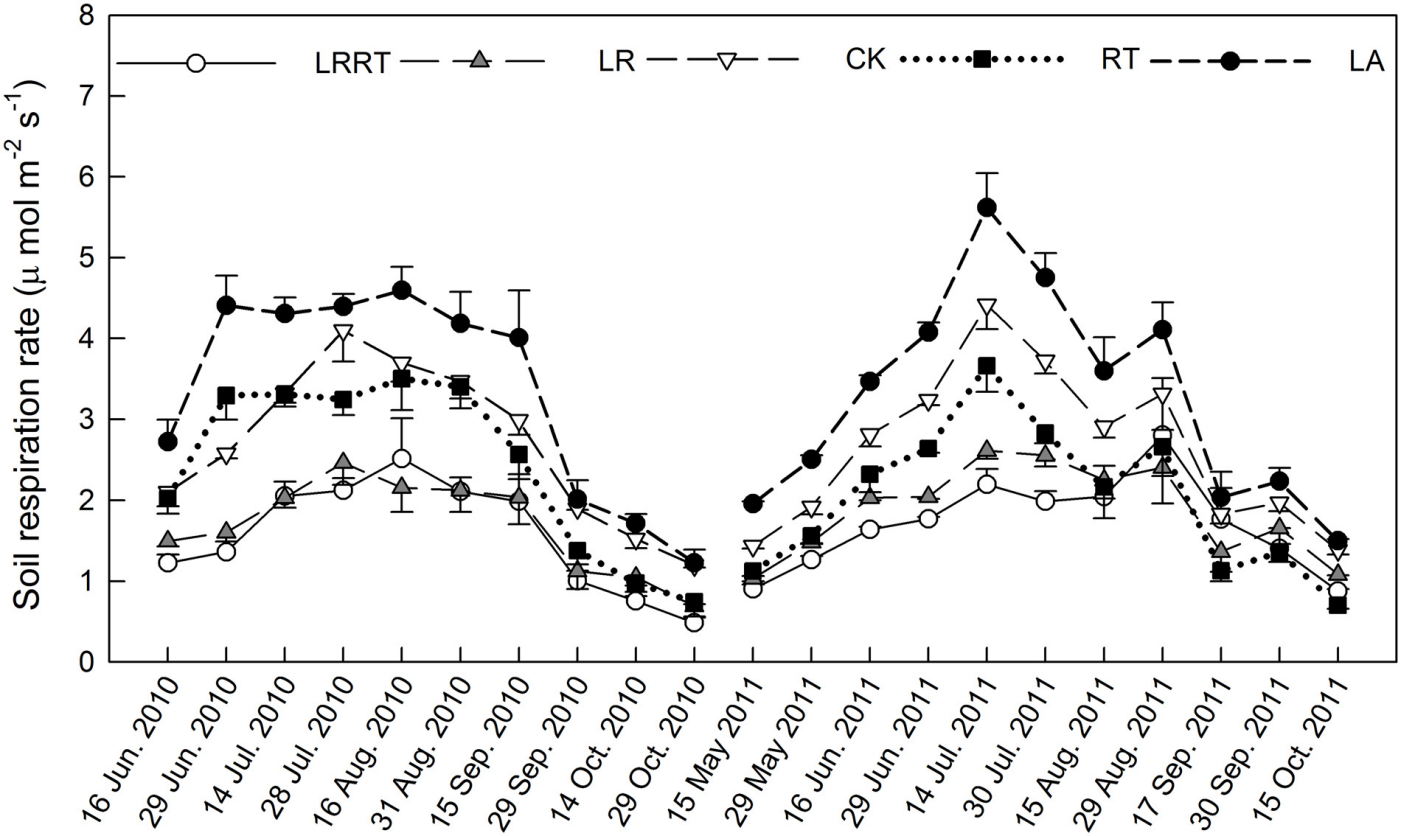
Variations of soil respiration in the various organic matter treatments as a function of time.

Soil respiration rate exhibited clear seasonal variations in the various organic matter treatments (Fig 2). Maximum *R*_s_ occurred during the summer, due to high temperatures and moisture in the soil, with minimum *R*_s_ in October, when mean monthly precipitation was low.

Soil organic matter treatments significantly affected *R*_s_ throughout the study period (*p*<0.001) (Table 1). The average *R*_s_ were 1.62, 1.82, 2.73, 2.30 and 3.42 μ mol m^-2^ s^-1^ in the LRRT, LR, CK, RT, and LA treatments, respectively. Either aboveground litter removal or root trenching significantly decreased *R*_s_ when compared to the CK (*p*<0.001; Table 2).

**Table 1:** Two-way analysis of variance (ANOVA) results of soil respiration rate (*R*_s_), soil temperature (*T*) and soil moisture (*M*) at 5 cm depth.

Source of variation	*R*_s_		*T*		*M*	
	*F*	*p*	*F*	*p*	*F*	*p*
**Season (*S*)**	0.009	0.926	1.413	0.235	4.113	0.05
**Treatment (*Tr*)**	54.048	0.000	1.537	0.190	16.706	0.000
***S*×*Tr***	1.216	0.303	0.109	0.979	2.049	0.086

**Table 2:** Two-way analysis of variance (ANOVA) results of soil respiration rate (*R*_s_), soil temperature (*T*) and soil moisture (*M*) at 5 cm depth during the entire observation period (aboveground litter and root trenching being the main factors).

Source of variation	*R*_s_		*T*		*M*	
	*F*	*p*	*F*	*p*	*F*	*p*
**Aboveground litter effect (*ALE*)**	82.879	0.000	2.360	0.125	4.985	0.05
**Root trenching effect (*RTE*)**	13.254	0.000	0.093	0.760	29.826	0.000
***ALE*×*RTE***	1.788	0.182	0.995	0.319	3.671	0.056

**Fig 3:**
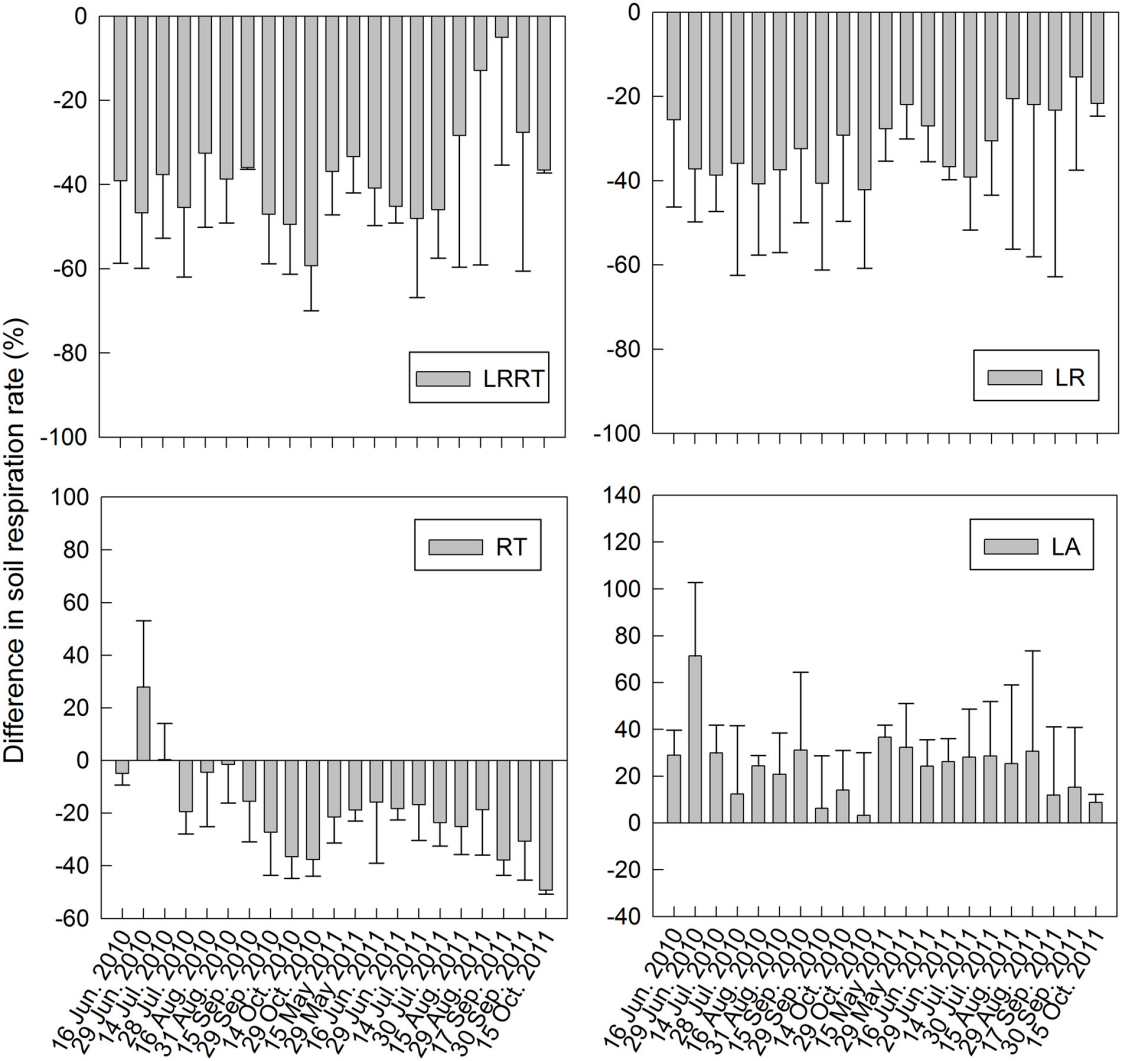
Changes in soil respiration rates in the various organic matter treatments as a function of time. Bars represent standard errors.

On average, *R*_s_ was significantly reduced by the LRRT treatment, about 38.93% ± 2.01% lower than in the CK (Fig 3). The corresponding values in the LR and RT treatments were 30.65% ± 1.87% and 17.65% ± 1.95% lower than in the CK. The soil respiration rate in the RT treatment was initially higher than that of the CK for up to 2 months after trenching, with rates, in late June and mid-July 2010, 27.87% and 0.33% higher than that of the CK. Soil respiration rate was significantly increased in the LA treatment, about 25.82% ± 2.44% higher than in the CK.

### Contribution of soil respiration components

**Fig 4:**
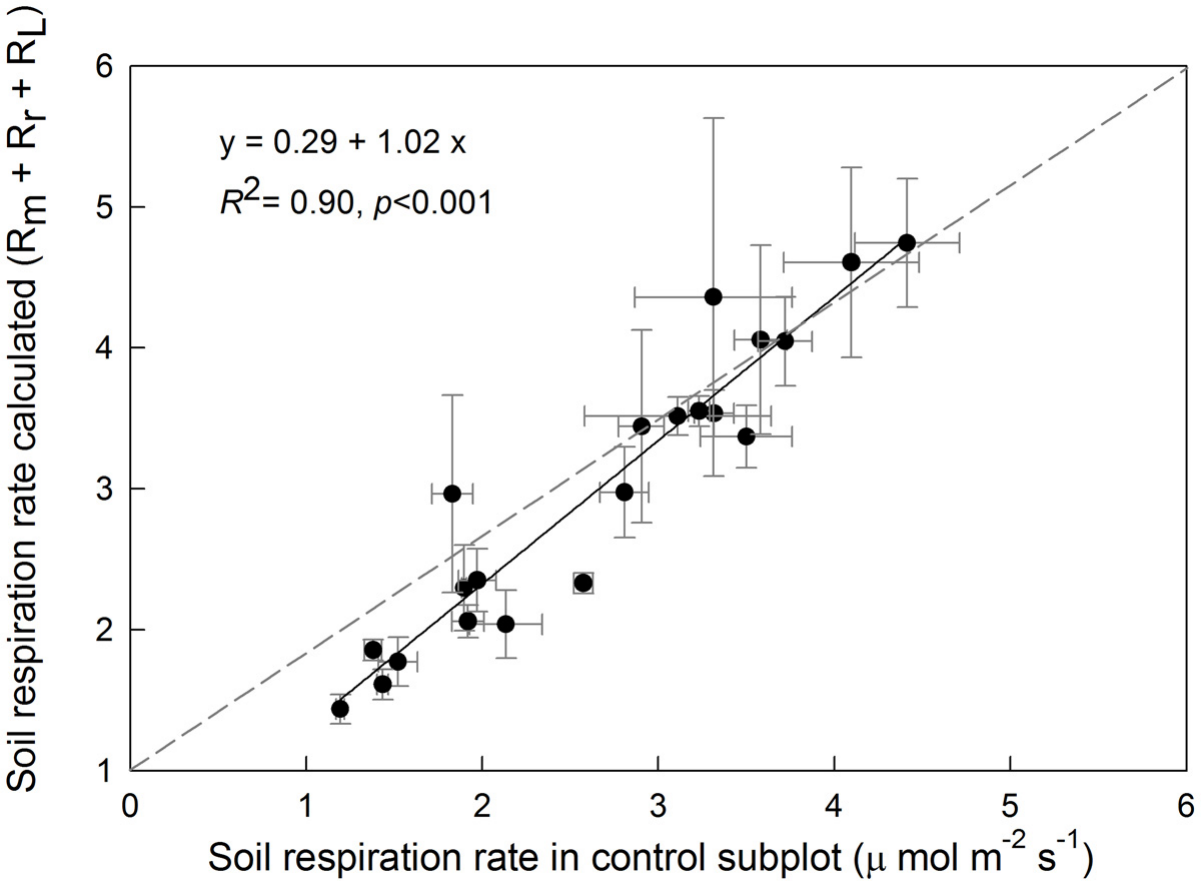
Relationship between soil respriation rate in the control subplots and the caluculated value as the sum of the different components. Bars represent standard errors. Dark gray line is 1:1 line and black line is fitting line.

On average, *R*_m_, *R*_L_ and *R*_r_ were 1.61, 0.93 and 0.46 μ mol m^-2^ s^-1^ during the entire observation period. The relative contribution of each component to total soil respiration rate was 53.7%, 31.0% and 15.3%. The sum of each component was calculated and compared with the soil respiration rate in the CK. The slope of the fitted equation of the calculated respiration rate as a function of the respiration rate in the control subplots, i.e. b_1_ = 1.02 is not significantly different from 1 (*p* = 0.85) (Fig 4).

### Comparison of accumulative seasonal soil respiration efflux

The accumulative seasonal soil respiration efflux varied between 241.0 and 532.5 g C m^-2^ in 2010, with the accumulative seasonal soil respiration amounts in 2011 ranging between 300.8 and 582.4g C m^-2^ (Table 3). The accumulative seasonal soil respiration in the LRRT and LR treatments was significantly lower while the value in the LA treatment was significantly higher relative to that of the CK (*p*<0.05). The RT treatment significantly decreased the accumulative seasonal soil respiration compared to the CK in 2011 (*p*<0.001); however, there was no significant difference between the accumulative seasonal soil respiration in the RT treatment and that in the CK in 2010 (*p*>0.05).

**Table 3:** Accumulative seasonal soil respiration efflux (g C m^-2^) among different organic matter treatments during growing seasons (*GS*) of 2010 and 2011.

Season	Treatment				
	LRRT	LR	CK	RT	LA
**2010 *GS***	241.0 a	266.1 a	424.2 b	387.3 bc	532.5 d
**2011 *GS***	300.8 a	337.6 a	472.4 b	356.5 a	582.4 c
**Average**	270.9 a	301.9 a	448.3 b	371.9 c	557.5 d

Different letters within the same row indicate significant difference among organic matter treatments (Two-way ANOVA with LSD test, α = 0.05)

### Effects of organic matter treatments on biophysical factors and microbial biomass carbon in the soil

Soil temperature showed distinct seasonal variations in the various organic matter treatments. During the entire observation period, no significant differences in soil temperature were found between the five organic matter treatments (*p*>0.05; Table 1). The average soil temperature was 13.4°C in the LRRT, 14.0°C in the LR, 12.8°C in the CK, 13.1°C in the RT and 12.6°C in the LA treatment throughout the study period.

Soil moisture also experienced clear seasonal variations in the various organic matter treatments. Organic matter treatment significantly affected soil moisture over the course of both growing seasons (*p*<0.001). The average soil moisture was 29.89% in the LRRT, 23.68% in the LR, 23.41% in the CK, 26.39% in the RT and 20.75% in the LA treatment throughout the study period. Soil moisture in the LRRT treatment was significantly higher than that in the CK (*p*<0.001). No significant differences in soil moisture were found between the other four treatments (*p*>0.05).

Organic matter treatment significantly affected microbial biomass carbon (MBC) in the soil (*p*<0.05). The average MBC was 110.9, 171.1, 240.5, 189.9 and 301.0 mg kg^-1^ in the LRRT, LR, CK, RT and LA treatment, respectively. MBC in the LA treatment was significantly higher while the values in the LRRT, LR and RT treatments were significantly lower than that of the CK (*p*<0.05).

### Effects of soil temperature and soil moisture on soil respiration

Correlations between soil temperature or soil moisture and soil respiration rate were significant in all treatments during the entire observation period (*p*<0.001; Table 4), with *R* ^2^ values ranging from 0.125 to 0.763. A significant quadratic relation of *R*_s_ to soil moisture was found across all treatments (*p*<0.05). We found that soil moisture explained 6.3%–17.2% of the variation in *R*_s_ throughout the study period. *Q*_10_ values based on the exponential regression of the Eq 2 are higher in the RT (2.72) and LA (3.19) treatments relative to the CK (2.51) and lower in the LRRT (1.52) and LR (1.36) treatments. The non-linear model (Eq 4) including both soil temperature and soil moisture, predict *R*_s_ rather well, with *R* ^2^ values ranging from 0.597 to 0.829 (Table 4). With the combined effect of soil temperature and soil moisture, the fitted *Q*_10_ values of the Eq 4 were 1.92, 2.04, 2.51, 3.00 and 3.25 for the LRRT, LR, CK, RT and LA treatment, respectively.

**Table 4:** Parameters of different models of soil respiration rate (*R*_s_) as a function of soil temperature (*T*) and soil moisture (*M*) at 5 cm depth. Data are mean values, with the SE given in parentheses.

Treatment	Parameters					
	*T* model					
	*β*_0_	*β*_1_	*p*	*R* ^2^	*Q*_10_	
**LRRT**	0.952 (0.122)	0.042 (0.008)	0.000	0.235	1.52	
**LR**	1.308 (0.172)	0.031 (0.008)	0.000	0.125	1.36	
**CK**	0.795 (0.083)	0.092 (0.007)	0.000	0.680	2.51	
**RT**	0.582 (0.070)	0.100 (0.008)	0.000	0.703	2.72	
**LA**	0.738 (0.078)	0.116 (0.007)	0.000	0.763	3.19	
	***M* model**					
	***β***_**2**_	***β***_**3**_	***β***_**4**_	***p***	***R*** ^**2**^	
**LRRT**	2.007 (1.105)	–0.081 (0.085)	0.002 (0.002)	0.000	0.172	
**LR**	1.276 (0.478)	0.029 (0.045)	–0.0002 (0.001)	0.05	0.063	
**CK**	0.572 (0.552)	0.200 (0.052)	–0.004 (0.001)	0.001	0.141	
**RT**	–0.695 (0.891)	0.227 (0.074)	–0.004 (0.002)	0.001	0.116	
**LA**	1.485 (0.722)	0.220 (0.076)	–0.005 (0.002)	0.05	0.082	
	***T & M* model**					
	***β***_**5**_	***β***_**6**_	***β***_**7**_	***p***	***R*** ^**2**^	***Q***_**10**_
**LRRT**	0.085 (0.386)	0.065 (0.006)	0.590 (0.111)	0.000	0.597	1.92
**LR**	0.165 (0.211)	0.071 (0.005)	0.433 (0.057)	0.000	0.691	2.04
**CK**	0.477 (0.121)	0.092 (0.005)	0.162 (0.035)	0.000	0.811	2.51
**RT**	0.322 (0.225)	0.110 (0.006)	0.127 (0.069)	0.000	0.807	3.00
**LA**	0.786 (0.121)	0.118 (0.005)	–0.032 (0.036)	0.000	0.829	3.25

## Discussion

### Response of soil respiration to organic matter manipulation

The findings from our manipulative experiment provide an insight into the effects of above- and belowground organic matter on soil respiration in a *P*. *tabulaeformis* plantation and may have significant implications in modeling soil respiration. Organic matter manipulation is expected to affect soil respiration by altering microclimatic conditions [37, 38], carbon chemistry [39] and microbial biomass in the soil [40]. There are many dimensions to organic matter which affect soil respiration, i.e., the type of organic matter, the amount of organic matter and timing of measurement.

Soil respiration includes autotrophic and heterotrophic respiration fluxes [41], showing different responses to organic matter manipulation. Autotrophic respiration is controlled by the root biomass of a specific soil layer, whereas heterotrophic respiration depends on the amounts of aboveground litter and dead organic carbon in the soil [42]. Despite some differences in soil respiration responses to aboveground litter removal and/or root trenching during the first 2 years, we found that either aboveground litter removal or root trenching both decreased soil respiration rate. This is mainly due to aboveground litter removal and root trenching, which leads to a lower carbon and nutrient supply from the aboveground to belowground layers and from roots into the soil, with a consequent lower microbial activity. Our results are also in line with the study conducted by Li et al. [21], who reported that litter removal decreased soil microbial biomass by 67–69% in a tropical pine plantation seven years after the initiation of treatments.

As predicted, litter removal had a higher impact than root trenching on soil respiration throughout the entire observation period. We found that soil respiration rate in the RT treatment did not decrease as expected but increased in late June and mid-July in the first year, which was probably due to the fact that roots are able to maintain respiration for a period of time after trenching. In addition, microbial decomposition of dead roots in the trenching subplots might provide substrates for the growth of microorganisms and stimulate soil respiration rate by increasing heterotrophic respiration [43]. Root trenching also increased soil temperature and soil moisture when compared to the control subplots. This may in turn stimulate soil respiration rate. After all, maintenance respiration, stimulation of heterotrophic respiration and changes in microclimatic conditions after trenching are possible causes for the initial increase of soil respiration rate in the RT treatment.

Although we did not observe any significant differences in the accumulative seasonal soil respiration between the RT treatment and the CK in 2010, the accumulative seasonal soil respiration in the RT treatment was significantly lower than that in the CK in 2011. This implies that root trenching result in changes in soil respiration that varies over time. According to Lee et al. [44], a short initial increase (of about 2 months) is followed by two years of decrease in soil respiration rate in a cool-temperate deciduous forest. Therefore, the increasing long-term and year-round measurements over time should be given more attention in future studies.

It has been reported that soil respiration rate always increases disproportionately in response to litter addition [45]. Litter addition not only releases a portion of the newly added carbon, but also accelerates the decomposition of older organic matter through the positive priming effect. For example, Prévost-Bouré et al. [45] reported that fresh aboveground litter addition over-stimulates soil respiration in a temperate deciduous forest owing to this positive priming effect. Moreover, this priming effect lasted for more than one year in the progressive decomposition of fresh litter. Contrary to our prediction, we did not find such a positive priming effect in the present study. The possible causes may be attributed to two factors. In first instance, litter addition in our study was only applied once at the beginning of the experiment, whereas other studies added fresh litter several times per year [22,25], which made the priming effect to be released gradually. Secondly, the depth of the litter layer in our pine plantation was up to 10 cm thick and needle litter decomposition was much slower than that from most broad-leaved forests [20,46]. Therefore, there were no significant differences between the increase in soil respiration rate in the LA treatment and the decrease in soil respiration rate in the LR treatment.

### Effects of soil temperature and soil moisture on soil respiration

It is well known that seasonal changes in soil respiration rate have been widely reported to be correlated with soil temperature and soil moisture [34,42,47]. Since our manipulative experiment was conducted in a relatively small area and most importantly, crown closure in our plantation did not change, organic matter manipulation did not significantly affect soil temperature in this study. However, the mean soil moisture in the LRRT treatment significantly increased compared to the CK throughout the entire observation period due to the inhibition of water transport between the trenching subplot inside and outside. Such a change would lead to corresponding changes in soil respiration rate.

The variation in soil respiration rate in our organic matter treatments was more sensitive to change in soil temperature than that in soil moisture during both growing seasons. It is probable that much of the soil moisture was in a range suitable for soil respiration, whereas soil temperature, kept at a low level during the study period, became a major factor restricting soil respiration rate. Soil temperature explained 68.0% to 76.3% of the seasonal variation in soil respiration rate, except in the LRRT and LR treatments. Soil temperatures in the LRRT and LR treatments were higher, but the respiration rates lower relative to the CK. This suggests that when aboveground litter is removed, soil temperature is still important, but not as sensitive as before to the seasonal variation in soil respiration rate. Aboveground litter acts as a protective buffer against air temperature. Therefore, the large variation in soil temperature after litter removal is not sufficient in explaining the variation in soil respiration rate.

The sensitivity of soil respiration to soil temperature in the CK was within the reported range (*Q*_10_ = 1.8–4.1) worldwide [48] and close to the reported median value of 2.4 studied by Raich and Schlesinger [49]. The *Q*_10_ values also varied among organic matter treatments in the present study. It has been reported that higher temperatures increase soil respiration efflux to the atmosphere, thus further aggravating global warming [50]. The highest *Q*_10_ value in the LA treatment suggests that litter addition enhances the sensitivity of soil respiration to changes in soil temperature and that soil respiration efflux in the LA treatment could be increased more under climate warming. Soil respiration is not significantly affected by climate warming in the LR treatment. It is caused by that leaf litter decomposition is more sensitive to soil temperature than root litter decomposition. Another reason is that after leaf litter is removed from aboveground, less organic carbon is transferred into soil. In our study, the bivariate model yielded higher *Q*_10_ values than the univariate model alone. It is possible that the relationship between soil respiration and soil temperature is confounded by soil moisture, given that we modified the relationship when soil moisture was considered.

## Conclusions

This study is the first report showing the effect of organic matter manipulation on soil respiration in a major pine ecosystem in temperate China. The results of this study will enhance our understanding of the complex impact of above- and belowground organic matter on soil respiration and thus on the ecosystem carbon budget. Either aboveground litter removal or root trenching caused a decrease of soil respiration rate while litter addition increased soil respiration rate over the 2-year period. Litter removal had a higher impact than root trenching on soil respiration throughout the entire observation period. The mean rate of soil respiration in the LR treatment was 30.65% ± 1.87% lower than in the CK, whereas this value in the RT treatment was 17.65% ± 1.95% lower than in the CK. When aboveground litter was removed in the LRRT and LR treatments, soil temperature was not as sensitive as before to the seasonal variation in soil respiration rate. Soil temperature and soil moisture were the main controlling factors of the seasonal variation in soil respiration rate. Up to the 59.7% to 82.9% seasonal variation in soil respiration is explained by integrating soil temperature and soil moisture in the various organic matter treatments.

Our current results suggest that litter addition will increase soil respiration efflux in response to climate warming, while litter removal will decrease the sensitivity of soil respiration to changes in soil temperature. Litter layer removal and understory clearing are common forest management practices in many countries and regions. Appropriate forest management in forest ecosystems emphasizes the promotion of organic carbon turnover and carbon sequestration in the soil. Therefore, our finding is crucial to forest managers in both predicting the consequences of forest management and guiding to manage forest carbon flux in temperate pine plantations. In temperate China, there are also large areas of natural pine forests, where *P*. *tabulaeformis* grows with a mixture of various broadleaved species. We speculate that soil respiration responses to organic carbon manipulation in natural forests would be different from that in plantations due to variations in the amount and quality of organic carbon and microclimatic conditions. To clarify the differences between natural forests and plantations would be critical to the management of regional carbon fluxes of *P*. *tabulaeformis* ecosystems. Further studies focusing on natural forests of *P*. *tabulaeformis* are therefore needed. In addition, as the responses of soil respiration to organic matter manipulation may vary over time, increasing long-term and year-round measurements over time should be given more attention in future studies.
